# Notes of an European Tour

**Published:** 1845-06-01

**Authors:** F. H. Hamilton

**Affiliations:** Prof. of Surgery in Geneva Medical College


### Notes of an European Tour.

Communicated for the Buffalo Medical Journal, by F. H. HAMILTON, M. D., Prof. of Surgery in Geneva Medical College.

We are happy to inform our readers that Prof. Hamilton will contribute regularly to our pages, and that the following is the first of a series of communications, giving an account of such incidents and observations pertaining to his European tour, as will be interesting to the profession. The series may be expected to extend, at least, over the numbers of the Journal for the curr nt year. We think an apology for the non-medical character of the first number, is quite gratuitous. Our readers, we are sure, will not be so hyper-critical as to insist that our friend should write nothing but what is redolent of medicine; but will be well pleased to accompany him on his route to Paris, before entering upon topics of a more professional character.—*Editor.*

Dr. Flint—*Dear Sir*—The object of my tour made last year, some notes of which, originally addressed to a friend, I propose to give you, was to visit the Medical Colleges, and the various Hospitals of Europe; and, also, to collect Anatomical, Surgical and Pathological specimens for our College Museum. I journeyed, therefore, through England, Ireland and Scotland; France, Belgium, Holland, Germany, Switzerland, Italy and Sicily; visiting, everywhere, the principal Hospitals, Museums, &c. I have commenced with France, because my tour of the British Isles, having been made at two separate dates, I thought to give it you entire, at the last. You have requested me to preserve the integrity of my notes, even at the hazard of introducing some links which are not Medical, that the whole may serve as a sort of "Guide book of Travel," to such of your readers as may wish to visit Europe for a similar purpose. But how much of Medicine or Surgery can one find between Boulogne and Paris? Yet. I trow, the English valetudinarian, suddenly transferred from an English coach and roads, to a French diligence and French roads, might find much of both—but a man whose bones will hold together upon a Western "corduroy," has little to fear from the square stone blocks of a Continental turnpike. Yours, truly,

*Buffalo, May* 12, 1845. F. H. Hamilton.

Dear M——, I was weary of the smoke and noise of London ; and I thought it best to hasten my southern tour, lest I should find the season too far advanced for either comfort or health. Accordingly, on Wednesday I packed and came on board the steamboat "Harlequin," for Boulogne. The steamer did not leave until Thursday morning, but at so early an hour—3 o'clock—that passengers were obliged to be on board at night. In the morning, when I went on deck, we were some fifty miles from London, under a most lovely sky, and were making about twelve miles an hour. The Thames had now opened into a broad frith, and at short distances along its banks were beautiful country villas, occupied mostly by the nobility and gentry of London, during the warm season. On the right we passed "Reculvers" or the "Two Sisters ;" two towers with turrets, rising from the brow of a lofty cliff. They are the remains of an ancient stone Church, the main building having long since fallen away from the encroachments of the sea. Next, we passed Margate, standing upon the same line of bold, chalky cliffs. Farther on, is Ramsgate and Deal, the latter defended by a strong castle, near which, and extending several miles, are the Downs, filled with vessels from all nations, at anchor, waiting for the tide. We were now fairly within the Straits of Dover, and our course lay close under the old Castle of Dover, which, as seen from the vessel, seemed impregnable ; no less so indeed, than the cragged rocks from which it arose, and of which it might be but a part. It was here poor blind Gloster would have ceased his miseries.

Glo.—"Dost thou know Dover?"
Edg.—"Ay, Master "
Glo.—" There is a cliff, whose high and bending head,
Looks fearfully in the confined deep;
Bring me but to the very brim of it."

A light fog was resting upon the eastern coast, and Calais could not be seen; yet, the mate told me, that on a clear day it was always visible, the distance being 24 miles. We were now 36 miles from Boulogne, and I have never seen the ocean so perfectly quiet as in the whole remainder of my passage. I had been told the channel was generally rough, but it was to-day scarcely moved by a ripple.

At Boulogne-sur-mer, we entered a small harbor, made by the extension of two substantial piers into the channel, and at 2 o'clock P. M. I first placed my foot upon the continent of Europe. The Custom House was close upon the wharf, and after delivering my passport, answering to my name, age and residence, and uncovering my head to enable the officer to sketch my portrait, I was permitted to pass. The diligence for Paris did not leave until 4, which gave me a little time to "look about." If, on landing at Liverpool, everything had seemed new and strange, here everything seemed more new, and more strange. Curious old buildings, with streets, narrow, and without sidewalks, and with names all in keeping —such as *La Rue tant perd tant paie*, or, as I translate it, *The street in which you will loose all you pay*—*La Rue du Puits d'Amour*—*Street of the Sources of Love*, &c. Everywhere, the women were running about the streets, without bonnets, with only neat, pretty caps over their neat, pretty faces, and chattering like jackdaws. Understand them? Perfectly: not that I am a scholar in French, yet they were so animated—their eyes sparkled so brightly—and their faces were so illumined when they laughed, and they laughed constantly—that, like one mesmerised, I could see and read all they thought. There is no language like the language of expression! It is the only universal language, and the French are its only masters. It is, perhaps, a curious phenomenon, that then, for the first time in my life, among a people of a strange world, strange manners, and strange tongue, I yet felt more at home than at any time since I had left America. If one would love the French, let him first go to England. They are antipodes———In France, every man, woman and child, old and young, soldier, priest and peasant, wherever or whenever you meet them, seem to give you a welcome—a sunny welcome, which you recognize before they speak, and which warms and attracts you insensibly. Have Physiologists ever determined, what influence a damp and chilling air, like that of England, may have upon the character of a people?

Having, on my first arrival, given my keys to a "commissionaire," with orders to attend to passport, luggage, &c. I found, on returning to the "Post," that all was ready. The luggage had been examined, but no charge made for foreign books, as at Liverpool. A "provisional" passport was given to me in place of the original: this was to serve me to Paris, at which place it is restored upon the presentation of the "provisional" at the proper office.

That you may know "how changed" I am, I will read you my portrait, as given in my new passport. "*Age*****. *Taille* d'un metre, centimetres 70. *Cheveu*, brun. *Front*, ardent. *Sourcils*, brun. *Yeaux*, brun—*nez*, moyen. *Bouche*, moyen. *Barbe*, brun. *Menton*, rond. *Visage*, ovale" (getting fat you see.) "*Teinte*, ardent."

At four I was mounted in the "Banquette" of a "Diligence." How shall I describe to you this curious vehicle! Imagine a prodigious omnibus, the roof, by full two feet, wider than the floor; a large, calash, carriage top, placed above and in front; with all the space behind the calash, and above the coach, filled with baggage for 16 or more, covered with a large oilcloth; and the whole of this unseemly bulk, placed upon four, heavy, lumber wheels—and you have some notion of a "Diligence."—There are four classes of seats, the coupe, interieur and rotonde, being the three divisions of the body of the Diligence; while the banquette, is above and back of the driver, under the calashe—the cheapest and the best seat in pleasant weather, being airy and out of the dust, and affording the best opportunity to see the country. Six horses, in the most primitive harness, three in each set of traces, is the usual number; but, occasionally, on ascending a hill, one or two more are added. The "Conductor," a very civil fellow, in a livery of a blue cap and corded roundabout, sits in the banquette, and the driver in front. A Normandy driver would amuse you; he is never idle, but urges, curses and goads his lumbering horses without cessation, and you will hear every now and then his favorite oath, "sacra, sacra, nomme de Dieu." The roads, all the way from Boulogne to Paris, are wide, level, and paved in the centre with square blocks of stone. In all these old countries, public highways are broad, and well made; but the streets of cities and villages, are narrow and inconvenient. The highways are the property of the government, and the horses belong to the Postmasters, who buy their appointment from the Government. Independent companies own the Dilligences, and must hire their horses from the Postmasters, the number at each Post being regulated by law. In this part of France, the horses are remarkably strong and heavy, but seldom possess speed. An opposition is now running on this same route, and as by law no Diligence can pass another except at a "Post," while horses are changing, the great struggle is at these points. The horses are changed by half a dozen or more men, women and boys, with extraordinary dispatch. I could no more than dismount from my roost, before I would hear the conductor, "montez, Monsieur, montez," and off we went again like a shot, ratling and tumbling, and thundering over the stones, the driver shouting, and cracking the long gad, sometimes, both driver and conductor, plying at the same time. Then the beggars! at every hill where our pace was a little slackened, were boys and girls, old men and old women, seldom looking poor, or dirty, or ragged, but French beggars, healthy, rosy faced and neat, with pretty bouquets in their hands, crying, in the most musical, plaintive tones, "charite, si vous plais, monsieur, oh! charite, si vous plais," laughing while they cried.

The whole country from Boulogne to Paris, is beautifully undulating, and covered with fields of wheat, rye, oats, trefoil, corn, potatoes, and rape; the oil, extracted from the seeds of which, is here in great use. Beets, are also raised in great abundance, for the manufacture of sugar. No fences are seen, except, rarely a hedge, or a plastered wall. Female peasants are here and there, some feeding their cattle by the roadside, keeping them from the improved fields by long ropes attached to their horns, or, patiently, attending their small flock of sheep, assisted by the faithful dogs, who, with an almost instinctive intelligence, restrain them from the least encroachment upon the great man's demesne.

A few miles from Boulogne we passed the dirty hamlet of "Pontbrique," near which Napoleon reviewed his troops when preparing to invade England. The house in which he was quartered is still shown. It was night when we entered Montrieul, an old walled and strongly fortified city, built upon a rocky eminence, surrounded by an artificial ditch, and an extensive, marshy plain. As we approached, the moon was shining full upon its heavy battlements, and its irregular pile of towers and stone buildings, which rose high above the line of the outer walls. We awoke the sleeping guard at the drawbridge and entering through a massive gateway, wound up a long and rather steep ascent, and through narrow streets, to the Post. Montrieul, being a frontier town, has often sustained severe and protracted sieges, and it is considered by the present king a very important military post.

A little beyond Montrieul we passed the forest of Crecy, where was fought, in 1346, the famous battle between the English and French, in which Edward, the Black Prince, then but a boy, greatly distinguished himself, and in which the force of the English as compared with the French, was as 1 to 8. This was the first considerable affair in which artillery was used; the English having in their possession a few ill-constructed cannon. From this battle, therefore, we may date those great revolutions in the art of war, which the employment of firearms has made; and, I doubt not, to a large extent, we may trace from hence the great improvements in the art of Surgery, to which war has always been subservient, and to which our Army Surgeons, during the last few centuries, have furnished such ample contributions. But few of the English fell at Crecy, but, of the opposing forces, on this and the succeeding day, nearly 60,000 were slaughtered, including 2 kings, 11 princes, and 100 nobles.

Abbeville, about 50 miles from Boulogne, situated upon the Somme, we passed in the night; and, more asleep than awake, I only remember entering through its gloomy walls, and rattling through narrow streets, lighted with, here and there, at long intervals, a lamp suspended over the middle of the way by a wire stretched across; and that the lights grew more and more dim, and the quaint old houses less and less distinct. When I awoke it was broad day light, and the old town of Abbeville was far behind us.

Passing through many smaller towns we reached Beauvais about 9 o'clock. This town, situated on a small stream, the Therain, is celebrated as having been successfully defended in 1472 against 80,000 troops under "Charles the terrible," by the women of the place headed by Jeanne Hachette. An annual procession, headed by the women, still takes place in honor of their intrepid leader. Soon after passing Beauvais, we began to see vineyards, this being lat. 49½, their utmost northern limit for France.

The last considerable town, before we reached St. Dennis, was Beaumont, upon the Therain. It presents nothing of interest. But shortly after leaving Beaumont, the conductor pointed to a chateau, on a hill, to the left, as that which belonged to the late Prince Bourbon Conde. The Prince had for many years lived here with the wife of the Baron de Feucheres, formerly Sophia Dawes, but not many years since he came to a sudden and mysterious death. Before his death he had bequeathed this estate to one of the younger sons of Louis Phillippe. The Baronness, herself, is since dead.

Beggars now began to multiply, indicating our approach to the great metropolis of wealth and luxury. Really diseased, lame, wretched and piteous looking beggars—such as I had seen about London and Liverpool; and such as you may seen everywhere, where kings and princes congregate—who suck up the wealth of the people like a sponge, and drain off even the sources of life and health. By an ordinance of the city of Paris, they are forbidden to beg within the city limits, because—they obstruct the doorways of palaces, and stand outside the gates of royal pleasure grounds! They mar the white garments of the Priests at the entrance of the churches, and disturb by their prayers the prayers of the rich! Their sightless eyeballs and their leprous limbs, are exposed at the corners, and they rest their profane and ungodly deformities against the sculptured marble, and sadly blot the beauties of nature and art! they awake disagreeable associations and unpleasant sympathies, and thus often contrive to levy a monstrous and unjust tax upon those who have robbed them! Was ever a nation and a king so oppressed? I have seen enough disease outside of Paris to require the daily attention of three surgeons. Are there not Hospitals enough in Paris? Doubtless. Yet these poor sufferers choose the chance of life which the open highway and the uncertain charity of the traveller offers, to that afforded by a Parisian Hospital. When more at leisure, I shall look for a reason—for these beggars too have *reason*, and will generally be found choosing and acting as other men.

Passing St. Dennis, and its splendid cathedral, the sepulchre of the French Kings, of which I shall write again, at 3 o'clock we stopped at the Barrier St. Denis de Paris, and having undergone a brief general inspection, and taken in the "octroi" officer, we drove direct to the Messagerie Generale, Rue St. Honore. Our trunks were now hastily examined, and at 5 o'clock I was comfortably quartered at 12 and 14 "Rue de Bussy"—not far from the "Ecole de Medicine," and in a part of the city most convenient for my pursuits.

